# Biogenic Amines in White Brined Cheeses

**DOI:** 10.3390/foods14030369

**Published:** 2025-01-23

**Authors:** Leona Buňková, Jakub Riemel, Khatantuul Purevdorj, Štěpán Vinter, Zuzana Míšková, Petra Jančová

**Affiliations:** 1 Department of Environmental Protection Engineering, Faculty of Technology, Tomas Bata University in Zlin, nám. T. G. Masaryka 5555, 760 01 Zlin, Czech Republic; bunkova@utb.cz (L.B.); j_riemel@utb.cz (J.R.); purevdorj@utb.cz (K.P.); vinter@utb.cz (Š.V.); 2 Department of Food Technology, Faculty of Technology, Tomas Bata University in Zlin, nám. T. G. Masaryka 5555, 760 01 Zlin, Czech Republic; miskova@utb.cz

**Keywords:** white brined cheeses, biogenic amines, lactic acid bacteria

## Abstract

In the current study, a comprehensive analysis of biogenic amines in white brined cheeses was conducted. BAs may accumulate in food in high concentrations via the activities of microorganisms that produce decarboxylation enzymes. Neither tryptamine, phenylethylamine, nor spermidine was detected in the monitored cheese samples. Biogenic amines were detected in 20 samples, with tyramine and spermine being the most abundant, particularly in Feta cheeses, where tyramine concentrations exceeded 100 mg/kg in three samples. In 25% of the tested cheeses, total concentration of all the monitored biogenic amines and polyamines exceeded the level of 100 mg/kg, which may be considered of toxicological significance to sensitive persons. Decarboxylase activity was identified in 94 isolates, including significant producers such as *Levilactobacillus brevis* and *Enterococcus durans*. The correlation between high total microbial counts and lactic acid bacteria suggests that these microorganisms play a critical role in biogenic amine production. Our findings highlight the importance of monitoring specific microbial populations to mitigate biogenic amine risks in cheese production.

## 1. Introduction

The presence of biogenic amines (BAs) in food industry products, especially in fermented foods like white brined cheeses, has received considerable attention due to their impact on food safety and human health. Biogenic amines are low-molecular-weight nitrogenous compounds formed primarily via the decarboxylation of amino acids by microorganisms during the fermentation process. Various factors can affect the accumulation of BAs in the context of cheese production, particularly with white brined cheeses. These factors include the choice of microbial strains, the quality of raw materials, and the specific fermentation conditions used [1,2,3].

White brined cheeses represent a category of dairy products, especially popular in Mediterranean and Balkan cuisines, characterized by their distinctive white color and high salt content, which is integral to their preservation and flavor profile. These cheeses, often referred to as “pickled cheeses”, are produced through the coagulation of various types of milk, including ovine, caprine, and bovine, followed by immersion in a brine solution containing 12–16% salt, which not only enhances flavor but also inhibits the growth of spoilage microorganisms and foodborne pathogens [4,5]. The group of white brined cheeses includes Feta, a traditional Greek cheese made primarily from sheep’s milk; Queso Blanco, a fresh cheese commonly used in Latin American cuisine; and Halloumi, a semi-hard cheese from Cyprus known for its suitability for grilling [6,7]. Other notable examples are Sirene, a Bulgarian cheese with a salty flavor, and Akawi, a mild cheese from the Middle East often used in pastries [4,8]. The sensory attributes and compositional characteristics of white brined cheeses can vary significantly based on cheesemaking practices, including the type of milk used, the method of salting, and the fermentation conditions [9,10]. The rapid pH decrease and lactic acid production during the initial ripening stages are crucial for inhibiting undesirable microorganisms [11]. Additionally, the ripening process involves complex biochemical changes, including proteolysis and lipolysis, influenced by the microbial communities present, leading to the development of unique flavor compounds [12,13].

While these cheeses are recognized for their nutritional benefits, including high protein content and potential antioxidant properties, it is essential to contextualize these benefits within the broader dietary framework. It is known that the high protein content of white brined cheeses contributes significantly to daily nutrient intake, particularly in protein-scarce diets [14]. However, it is also important to note that many cheeses are high in sodium, which can pose health risks if consumed excessively, thus warranting moderation in their consumption [15]. Additionally, the antioxidant properties attributed to certain cheeses can be linked to bioactive compounds such as polyphenols and peptides, which are influenced by the dietary habits of the animals from which the milk is sourced. For instance, cheeses made from the milk of animals fed diets rich in plant materials, such as olive leaves, have shown enhanced antioxidant activity due to the transfer of these beneficial compounds into the cheese matrix [15].

BAs in white brined cheeses, particularly histamine and tyramine, are critical to consider due to their potential health implications. Histamine is the primary concern, as it can lead to two distinct health issues: histamine poisoning and histamine intolerance. Histamine poisoning, commonly referred to as scombroid poisoning, occurs when foods containing high levels of histamine are consumed, resulting in symptoms such as flushing, headaches, and gastrointestinal distress [16]. This condition is often associated with fish but can also occur with certain cheeses that have elevated histamine levels due to microbial activity during fermentation. The European Food Safety Authority (EFSA) has noted that histamine levels above 100 mg/kg can lead to severe poisoning, while concentrations of 20 mg/kg are considered the threshold for clinical symptoms [17]. In contrast, histamine intolerance arises from an impaired ability to metabolize histamine, often due to reduced activity of the enzyme diamine oxidase (DAO), which can be exacerbated by consuming histamine-rich foods. Symptoms of histamine intolerance can mimic allergic reactions, including headaches, skin rashes, and gastrointestinal issues, but they are not mediated by an immune response [18]. Tyramine, while less frequently discussed, can also pose risks, particularly for individuals taking monoamine oxidase inhibitors (MAOIs), as it may lead to hypertensive crises [17,19].

Several studies have shown that the concentration of BAs in white brined cheeses can differ significantly based on various fermentation conditions, including temperature, pH, and the duration of fermentation [20]. The presence of specific lactic acid bacteria (LAB) strains is particularly influential; these microorganisms not only contribute to the formation of BAs but may also possess the ability to degrade them, thereby affecting the overall safety and quality of the cheese [21]. *Lactiplantibacillus plantarum*, *Latilactobacillus sakei*, and *Enterococcus faecium* are among the most notable strains. These LAB are known for their amino acid decarboxylase activity, which facilitates the conversion of amino acids into BAs such as histamine and tyramine during fermentation [22,23]. However, *Lactiplantibacillus plantarum* not only produces BAs but also exhibits the ability to degrade them, thereby reducing their levels in fermented products [24]. Additionally, *Enterococcus faecium* has also been recognized for its dual role in both producing and degrading biogenic amines, particularly histamine, making it a valuable strain for managing biogenic amine levels during cheese fermentation [25]. Furthermore, *Lactobacillus rhamnosus* has demonstrated effective histamine-degrading capabilities, which can help mitigate the adverse effects associated with high biogenic amine concentrations [26]. The EFSA has emphasized that the accumulation of biogenic amines, particularly histamine and tyramine, is closely linked to microbial action during fermentation, with LAB being significant contributors to this process [17,27].

The hygienic and sanitary conditions during production are critical, as poor hygiene can lead to contamination by spoilage microorganisms that further elevate biogenic amine levels [28]. Environmental factors, such as ripening conditions and the presence of free amino acids, also play a significant role in biogenic amine production, as they can enhance microbial activity and enzymatic processes, leading to the accumulation of these compounds [29,30]. The balance between the production and degradation of BAs is critical, as it determines the final amine profile of the cheese and its potential health implications [31,32].

Moreover, the microbial diversity present during the fermentation of white brined cheeses can affect the BA content to a large extent. The interaction between different microbial species can lead to a complex biochemical environment where certain strains may promote the accumulation of BAs while others may mitigate their levels [33,34]. This fact underscores the importance of selecting appropriate starter cultures and correctly managing fermentation conditions to minimize the risks associated with BAs in cheese production [35,36].

This paper investigates the presence of eight BAs in white brined cheeses sourced from various supermarket chains in the Czech Republic, specifically focusing on histamine, tyramine, tryptamine, putrescine, cadaverine, spermine, spermidine, and phenylethylamine. The selection of these BAs is based on their relevance to food safety and quality, as they are commonly associated with adverse health effects [37,38]. Histamine and tyramine are well-documented for their potential to cause toxic reactions in sensitive individuals, making their presence in cheese a significant concern [2,38]. In addition to monitoring BA, fundamental microbiological analyses are conducted, including the isolation and identification of microorganisms present in the cheeses. The decarboxylase activity of the isolates is also tested to assess their potential contribution to biogenic amine formation. By analyzing these BAs and characterizing microbial populations, this research aims to provide considerable insights into the safety profile of commercially available white brined cheeses and to highlight the importance of monitoring BAs to ensure consumer health and product quality.

## 2. Materials and Methods

### 2.1. White Brined Cheese Samples

A total of 30 samples of white brined cheeses were purchased from various supermarket chains in the Czech Republic. The specific selection of these cheese types was based on their popularity in the Czech market. The selected cheeses encompassed both counter-sold cheeses and those available in consumer packaging, ensuring a diverse representation of market offerings, and were categorized as follows: (i) Jadel, consisting of five unflavored samples; (ii) Feta, comprising three unflavored samples; (iii) Balkan cheese, which included eleven unflavored samples and one flavored variant; and (iv) Akawi cheese, featuring five unflavored and five flavored samples. Notably, the Akawi cheese samples were stored under strictly controlled conditions at a temperature range of 20–25 °C for one year prior to the analysis. Each cheese, represented by five samples (five parts of one cheese), was subjected to a comprehensive assessment of BA contents across the different types and varieties. The codification of the cheese samples was designated in the following manner: A, C, E, G, H, J, K—Balkan cheeses in consumer packaging; B, D, F, I, L—Balkan cheeses, counter-sold; M, N, O—Feta, counter-sold; P, Q, R—Jadel in consumer packaging; S, T—Jadel, counter-sold; Z, AA, BB, CC, DD—Akawi, unflavored; V, EE—Akawi, gyros flavor; W—Akawi, garlic flavor; and U, X—Akawi, tzatziki flavor.

### 2.2. Microbiological Analysis

Five grams of each cheese sample were accurately weighed and placed into a sterile plastic bag. In the next step, 45 mL of physiological saline solution at pH 7.1 [sterile solution of 0.9% (*w*/*v*) NaCl (PENTA, Chrudim, Czech Republic)] was added to the sample. The contents of the bag were then homogenized using a stomacher (Stomacher 400; Seward Ltd., Worthington, UK) at a medium speed for 5 min, ensuring the thorough mixing and dispersion of the sample. Following homogenization, a tenfold dilution series was prepared from the resulting suspension to facilitate subsequent microbiological analyses.

The total microbial count was determined according to the ISO 4833-1 (2013) standard [39] with the following procedure: a volume of 0.1 mL (dilutions 10^−1^–10^−6^) of the inoculum was applied to sterile PCA (plate count agar, HiMedia Laboratories, India) and cultivated at 30 °C for 24 h. The enumeration of LAB was performed following the ISO 15214 (1998) standard [40], where 0.1 mL (dilutions 10^−1^–10^−4^) of the inoculum was applied to MRS (De Man, Rogosa, and Sharpe agar, HiMedia Laboratories, Mumbai, India) for lactobacilli and M17 (M17 agar, HiMedia Laboratories, Mumbai, India) for lactic cocci. Cultivation on M17 agar was conducted at 30 °C for 24 h, while cultivation on MRS agar was conducted at 30 °C for 48 h. The presence of coliform bacteria was assessed according to the ISO 4832 (2006) standard [41], with 0.1 mL (dilutions 10^−1^–10^−3^) of the inoculum spread onto sterile VRBA (violet red bile agar, HiMedia Laboratories, Mumbai, India). The plates were then incubated at 37 °C for 24 h. The presence of enterococci was determined according to the ISO 6887-1 (2017) standard [42], where 0.1 mL (dilutions 10^−1^–10^−3^) of the inoculum was applied to sterile SBA (Slanetz and Bartley agar, HiMedia Laboratories, Mumbai, India), and the cultivation was performed at 37 °C for 24 h.

Selected colonies were isolated in BHI (brain heart infusion, HiMedia Laboratories, Mumbai, India) broth, which is a rich, non-selective medium supporting the growth of a wide variety of microorganisms, and then cultivated for 24–48 h either at 37 °C for coliform bacteria and enterococci or at 30 °C for LAB and other microorganisms. Finally, t20% (*v*/*v*) sterile glycerol was added to the isolates, which were stored at −43 °C for further use.

### 2.3. Identification of Isolates with MALDI-TOF MS Biotyping

Selected colonies from the previous steps were further cultivated on TSA (tryptone soya agar, HiMedia Laboratories, Mumbai, India) to facilitate the growth of a diverse range of microorganisms. Each Petri dish was systematically divided into eight sections allowing the simultaneous cultivation of multiple isolates. A small volume of culture was transferred to each section using a sterile bacteriological loop, ensuring even distribution and minimizing cross-contamination. The inoculated Petri dishes were incubated at 37 °C for 24 h. After incubation, the isolated microorganisms were identified using MALDI-TOF MS biotyping (matrix-assisted laser desorption/ionization–time of flight), a sophisticated, rapid, and precise method for microbial identification based on the analysis of protein profiles. The identification was conducted immediately following the designated cultivation period. Colonies were selected based on the criterion that they had grown exclusively within their assigned sections and had not spread across multiple sections of the Petri dish.

A loopful of the grown colonies was collected using a sterile bacteriological loop and transferred to an Eppendorf tube containing 300 µL of ultrapure water. Next, 900 µL of absolute ethanol (99.8%, Sigma-Aldrich, St. Louis, MO, USA) was added to the suspension. The prepared sample was then centrifuged for 2 min at 14,000 rpm (MiniSpin plus, Eppendorf, Germany). After centrifugation, the supernatant was carefully discarded, and the same centrifugation procedure was repeated with the pellet. Any residual ethanol was removed, and the pellet was allowed to dry at room temperature. Subsequently, 10 µL of 70% formic acid (Sigma-Aldrich, St. Louis, MO, USA) was added to the dried pellet, which was then resuspended by pipetting and vortexing. Then, 10 µL of acetonitrile (Sigma-Aldrich, St. Louis, MO, USA) was added. The suspension was centrifuged at 14,000 rpm for 2 min. Next, 1 μL of the resulting supernatant was placed onto the MALDI target plate. Once the sample had dried, each spot was covered with 1 μL of HCCA (2-cyano-3-(4-hydroxyphenyl) acrylic acid) (Bruker Daltonics, Bremen, Germany) matrix and allowed to air dry at room temperature prior to analysis. The mass spectra were obtained using the MALDI-TOF method and processed with a Microflex LT instrument (Bruker Daltonics, Bremen, Germany) utilizing Flex Control 3.4 software along with Biotyper Realtime Classification 3.1 and the specific software for BC. Successful identification was completed based on a confidence score of ≥2.0 for species-level identification and ≥1.7 for genus-level identification, as specified by the manufacturer [43].

Appropriate biochemical testing kits were utilized to validate the identification results for the various bacterial isolate groups. Specifically, the ENTEROtest 24 (Erba Lachema, Brno, Czech Republic) was used for the identification of *Klebsiella oxytoca* and *Serratia marcescens*, while the STAPHYtest 24 (Erba Lachema, Brno, Czech Republic) was employed for *Staphylococcus* spp. and *Kocuria* spp. Additionally, the MicrogenTM Bacillus ID (Microgen Bioproducts Ltd., Camberley, UK) was utilized for the identification of *Bacillus* spp. These tests were selected due to their high specificity and sensitivity for the target organisms, critical for assessing microbial safety in food products. Each identification was performed in at least two independent experiments with two parallel tests conducted to ensure the reliability of the results.

### 2.4. Preparation of Samples and Precolumn Derivatization with Dansyl Chloride

The decarboxylase activity of individual bacterial strains was assessed after the strains were allowed to grow in a modified broth. The bacterial strains were cultured in BHI broth (HiMedia Laboratories, India) supplemented with specific amino acid precursors to the BAs and PAs being monitored for 24 h at 30 °C. Specifically, histidine, tyrosine, phenylalanine, ornithine, arginine, and lysine were each added at a concentration of 2 g/L (Sigma-Aldrich, St. Louis, MO, USA). After the cultivation, the contents of the tubes were centrifuged for 20 min at 4600 rpm (Jouan MR 23i, Jouan, Saint-Herblain, France). Following centrifugation, 750 µL of the supernatant was transferred into an Eppendorf tube. To this sample, 750 µL of perchloric acid (HClO_4_, Sigma-Aldrich, St. Louis, MO, USA) was added at a concentration of 1.2 mol/L. For each original sample, three Eppendorf microtubes were prepared in parallel, and these were stored at −18 °C until further analysis.

Lyophilized cheese samples were used for the analysis of BAs because the cheeses were purchased incrementally, and lyophilization allowed for their preservation and simultaneous analysis. A 1 g of lyophilized cheese sample was weighed into a tube, and 10 mL of perchloric acid (HClO_4_, Sigma-Aldrich, St. Louis, USA) at a concentration of 0.6 mol/L was added. Three parallel samples were prepared from each original cheese sample. The tube contents were mixed using a vortex mixer (Vortex Mixer VX-200, Labnet International, Inc., Edison, NJ, USA) and then shaken on a shaker (Orbital Shaker BS-1, Biosan, Riga, Latvia) for 30 min to facilitate thorough extraction. Following this, the mixture was centrifuged for 20 min at 6000 rpm (Jouan MR 23i, Jouan, Saint-Herblain, France). The resulting liquid fraction was transferred into a 25 mL volumetric flask (Simax, Prague, Czech Republic), and an additional 7 mL of 0.6 mol/L HClO_4_ was added to the flask. The contents were mixed again with a vortex mixer, shaken for another 30 min, and centrifuged for 20 min at the same speed. The liquid fraction was transferred once more into a 25 mL volumetric flask, and another 7 mL of 0.6 mol/L HClO_4_ was added. The sample underwent the same vortex mixing, shaking, and centrifugation process. After centrifugation, the final sample was transferred to a 25 mL volumetric flask and brought to the mark with 0.6 mol/L HClO_4_. Finally, the individual extracts were filtered through a membrane with a porosity of 0.45 µm to remove any remaining particles.

The following procedure was identical for both lyophilized cheese samples and microbial samples (cultivation broth). A volume of 100 µL of the internal standard (1,7 heptanediamine, Sigma-Aldrich, St. Louis, MO, USA), was added to each tube at a concentration of 500 mg/L. To this, 1 mL of extract, 1.5 mL of carbonate buffer (pH 11.0–11.1), and 2 mL of a freshly prepared solution of dansyl chloride (Sigma-Aldrich, St. Louis, MO, USA) in acetone (Sigma-Aldrich, St. Louis, MO, USA) at a concentration of 5 g/L were added. The tubes were then sealed and shaken in the dark for 20 h. Subsequently, 200 µL of proline solution (Sigma-Aldrich, St. Louis, MO, USA) was added, and the tubes were shaken for an additional hour in the dark. Following this step, 3 mL of heptane (Sigma-Aldrich, St. Louis, MO, USA) was introduced, and the samples were thoroughly shaken for 3 min. A volume of 1 mL from the heptane layer was then pipetted into a vial, which was subsequently evaporated to dryness at 60 °C under a nitrogen stream. To the dry residue, 1.5 mL of acetonitrile (Sigma-Aldrich, St. Louis, MO, USA) was added, and the vials were sealed and stored at −18 °C until analysis.

#### 2.4.1. Preparation of Carbonate Buffer

To prepare the carbonate buffer, 230 mL of solution A (0.5 mol/L, NaHCO_3_, Sigma-Aldrich, St. Louis, MO, USA) was combined with ca. 10 mL of solution B (0.5 mol/L, Na_2_CO_3_, Sigma-Aldrich, St. Louis, MO, USA). The pH of the resulting mixture was then adjusted to 9.2 by incrementally adding small amounts of solution B. Subsequently, 240 mL of the resulting solution was measured (amount sufficient for 156 samples). This buffer mixture remained stable for 7 days. Before proceeding with the derivatization process, 79.92 g of K_2_CO_3_ (Sigma-Aldrich, St. Louis, MO, USA) was dissolved in 240 mL of the prepared buffer (pH 9.2), resulting in an increase in pH to the range of 11.0–11.1. It is important to note that this carbonate buffer should be freshly prepared for optimal results during derivatization.

#### 2.4.2. Preparation of Dansyl Chloride at a Concentration of 5 g/L

A total of 1.6 g of dansyl chloride was weighed and placed into a beaker (Simax, Prague, Czech Republic), where it was then mixed with 320 mL of acetone.

### 2.5. Determination of Biogenic Amine Content

The concentrations of the eight monitored BAs, namely histamine (HIS), tyramine (TYR), phenylethylamine (PHE), tryptamine (TRY), cadaverine (CAD), putrescine (PUT), spermine (SPE), and spermidine (SPD), were quantified using high-performance liquid chromatography (HPLC) (Lab Alliance, State College, PA, USA and Agilent Technologies, Agilent, Palo Alto, CA, USA). Before analysis, the samples were filtered through a 0.22 µm syringe filter (Sigma-Aldrich, St. Louis, MO, USA) and injected into the chromatographic system. For separation, a Zorbax RRHD Eclipse Plus C18 column (50 × 3.0 mm, 1.8 μm, Agilent; Palo Alto, CA, USA) was used. Chromatographic separation was performed using a gradient elution method with the following solvent compositions: (A) acetonitrile (100%) and (B) acetonitrile (50%). The gradient was programmed as follows: from 0 to 2 min, the composition was set to A at 40% and B at 60%; from 2 to 3 min, A was increased from 40% to 80% while B decreased from 60% to 20%; from 3 to 4 min, A was adjusted from 80% to 90% and B from 20% to 10%; from 4 to 6 min, A was further increased from 90% to 95% and B was decreased from 10% to 5%; from 6 to 7 min, A was reduced from 95% to 40% while B was increased from 5% to 60%; and finally, from 7 to 12 min, the composition was maintained with A at 40% and B at 60%. The flow rate was maintained at 1.0 mL/min, with the column temperature set at 25 °C; a 5 μL sample injection volume was used, and detection was performed using a diode array detector set to a wavelength of 254 nm.

### 2.6. Statistical Analysis

The results of microbiological analysis were statistically analyzed using nonparametric tests, specifically the Kruskal–Wallis and Mann–Whitney tests. Additionally, Spearman correlation coefficients were calculated to assess the relationships between the total content of BAs and the total microbial count and the total content of BAs and LAB count. The analyses were conducted employing Unistat 10 statistical software (Unistat Ltd., London, UK), with the significance level set at 0.05 for all tests.

### 2.7. AI Tools

Scite AI was integrated to enhance the literature review process and streamline the identification of relevant studies. Scite AI employs advanced artificial intelligence algorithms to analyze and summarize scientific literature, allowing for the efficient extraction of pertinent information from various sources. By utilizing Scite AI, we were able to identify key studies that support our research objectives, including those related to the detection and quantification of BAs and/or PAs in food products. This tool facilitated the identification of primary literature and provided insights into how various studies have cited and built upon one another, thereby enriching our understanding of the current state of research in this field. It is noteworthy that the Scite AI license utilized for this research was funded by Tomas Bata University in Zlin, which demonstrates the institution’s commitment to supporting innovative research methodologies and enhancing academic resources for its researchers.

## 3. Results and Discussion

### 3.1. Occurrence of Microorganisms

**Figure 1:**
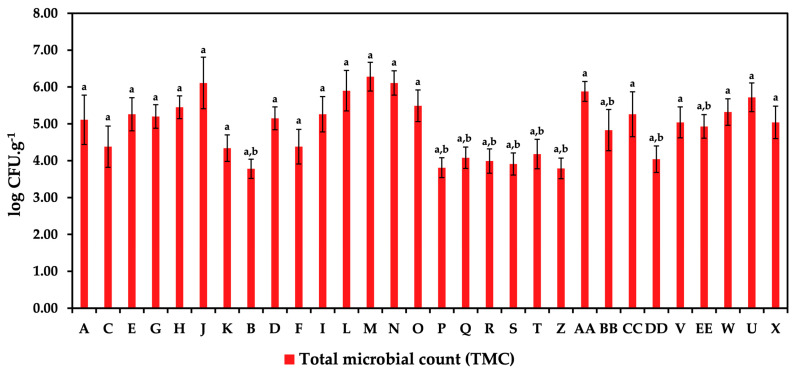
Total microbial counts in white brined cheeses. A, C, E, G, H, J, K—Balkan cheeses in consumer packaging; B, D, F, I, L—Balkan cheeses, counter-sold; M, N, O—Feta, counter-sold; P, Q, R—Jadel in consumer packaging; S, T—Jadel, counter-sold; Z, AA, BB, CC, DD—Akawi, unflavored; V, EE—Akawi, gyros flavor; W—Akawi, garlic flavor; U, X—Akawi, tzatziki flavor. Samples marked with the same letters show no significant statistical difference between them at a significance level of *p* < 0.05.

**Figure 2:**
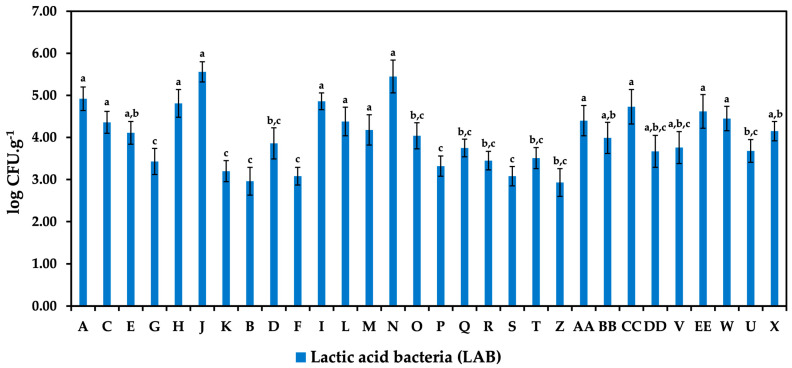
Lactic acid bacteria counts in white brined cheeses. A, C, E, G, H, J, K—Balkan cheeses in consumer packaging; B, D, F, I, L—Balkan cheeses, counter-sold; M, N, O—Feta, counter-sold; P, Q, R—Jadel in consumer packaging; S, T—Jadel, counter-sold; Z, AA, BB, CC, DD—Akawi, unflavored; V, EE—Akawi, gyros flavor; W—Akawi, garlic flavor; U, X—Akawi, tzatziki flavor. Samples marked with the same letters show no significant statistical difference between them at a significance level of *p* < 0.05.

This fundamental microbiological study analyzed 30 samples of white brined cheeses to identify their microbial quality. This analysis included the enumeration of TMCs, LAB, enterococci, and enterobacteria following the appropriate ISO standards for each category. The results of the TMCs and the specific counts of LAB are illustrated in Figure 1 and Figure 2. The highest TMC of 6.28 log CFU/g was recorded in sample M. This sample, identified as a counter-sold Feta cheese, exhibited the most substantial microbial load among the analyzed cheeses. In comparison, samples J and N, which were Balkan cheese in consumer packaging and counter-sold Feta cheese, respectively, demonstrated slightly lower counts of 6.11 log CFU/g. Notably, samples J and N also exhibited significant counts of LAB, with sample J showing a LAB count of 5.56 log CFU/g and sample N of log 5.45 CFU/g. Furthermore, considerable TMCs were observed in samples L (Balkan cheese, counter-sold), O (Feta, counter-sold), AA (unflavored Akawi), and U (tzatziki-flavored Akawi), with counts ranging from 5.49 log CFU/g to 5.90 log CFU/g.

Spearman correlation analysis was conducted to examine the relationship between the TMC and the total content of BAs. The analysis yielded a correlation coefficient of 0.23, suggesting a weak positive correlation. However, the *p*-value associated with this correlation was 0.33, which exceeds the established significance threshold of 0.05, indicating that the correlation is not statistically significant. Similarly, the relationship between LAB and the total content of BAs was analyzed, resulting in a correlation coefficient of 0.19. This value also indicates a very weak positive correlation; however, the *p*-value of 0.43, above the significance level of 0.05, confirms that this correlation is not statistically significant.

Elevated LAB levels contribute positively to flavor and texture development. On the other hand, many LAB strains are decarboxylase positive and can also lead to BA formation. The presence of significant LAB populations in the samples with higher TMCs indicates an environment conducive to BA production, particularly if specific LAB strains with decarboxylase activity are favored [2,44]. Previous research emphasizes the importance of monitoring microbial composition, as non-starter lactic acid bacteria (NSLAB) can dominate during ripening. Many NSLAB strains have relatively strong decarboxylase activity and can thus significantly increase the amount of BAs produced in cheese [9,45].

Enterococci were identified in 11 of the tested cheese samples. Specifically, enterococci were detected in Balkan cheese in consumer packaging (sample J), counter-sold Balkan cheese (sample F), all analyzed Feta cheeses (samples M, N, O), Jadel cheese in consumer packaging (sample R), and three unflavored variants of Akawi cheese (samples AA, CC, DD). Additionally, enterococci were found in flavored Akawi cheeses, including those with gyros flavoring (sample EE) and garlic flavoring (sample W). The presence of enterococci indicates their potential role in the fermentation process of white brined cheeses. The varying concentrations of enterococci, ranging from log 2.11 CFU/g to log 3.99 CFU/g, suggest that their metabolic activity may significantly influence the biogenic amine profile in these cheeses, potentially impacting both flavor development and safety. Due to their metabolic activity, enterococci are often key contributors to flavor development in traditional fermented dairy products. These bacteria are known to exhibit amino acid decarboxylase activity, which can contribute to the production of BAs, particularly tyramine and histamine, during cheese ripening [46]. The varying concentrations of enterococci suggest that their metabolic activity may significantly influence the BA profile in these cheeses, potentially impacting both flavor development and safety [47].

Enterobacteria were confirmed in only four of the analyzed cheese samples: Balkan cheese in consumer packaging (sample H), Feta cheese (sample N), Jadel cheese in consumer packaging (sample Q), and unflavored Akawi cheese (sample BB), with counts ranging from 2.32 log CFU/g to 3.38 log CFU/g. The detection of enterobacteria in only four of the analyzed cheese samples suggests a limited role in the overall microbial ecology of the cheeses. An environment with a higher concentration of NaCl is not very suitable for their growth. Other factors that may influence the presence of enterobacteria include hygienic conditions during cheese production, the use or omission of heat treatment for milk, and other processing steps. However, enterobacteria can influence biogenic amine production due to their potential decarboxylase activity, which may contribute to the formation of specific polyamines such as CAD and PUT during cheese ripening [48]. Although the counts of enterobacteria were relatively low, their metabolic activity may still pose a risk for BA accumulation, particularly in conjunction with other microbial populations present in the cheese matrix [2]. Therefore, monitoring enterobacteria levels is essential for managing BA risks and ensuring the safety and quality of white brined cheeses.

### 3.2. Biogenic Amines in White Brined Cheeses

BAs were detected in 20 samples of white brined cheeses, as shown in Table 1. Notably, none of the analyzed samples contained PHE, TRY, or SPD, indicating a limited diversity of BAs in these products. HIS occurrence was also low, being present in only one sample, sample M, at a concentration of 26.76 mg/kg. Another relatively low-abundance amine was CAD, identified in four cheeses (samples M, R, T, EE), with the highest concentration in sample M of 31.01 mg/kg. The observation that histamine was not detected at high concentrations is intriguing. This suggests the absence of histamine-producing strains or environmental factors unfavorable for their activity.

In the cheeses analyzed, TYR emerged as the most frequently detected BA, present in 18 samples. The highest TYR concentrations were found in Feta cheeses, specifically in sample M—380.43 mg/kg—followed by samples O—133.25 mg/kg—and N—122.71 mg/kg. In the remaining 15 cheeses, TYR concentrations varied from 2.38 mg/kg to 46.66 mg/kg. The second most prevalent BA was SPE, absent from only six of the twenty samples where BAs were confirmed, with concentrations ranging from 5.04 mg/kg to 20.97 mg/kg. Six samples exhibited elevated SPE levels, with concentrations between 40.39 mg/kg and 73.41 mg/kg. PUT was detected in only three of the tested cheeses, all of which were Feta varieties, with significant levels recorded in samples M—308.50 mg/kg—and N—349.99 mg/kg—while sample O exhibited a notably lower concentration of 12.27 mg/kg.

Most of the BA-containing cheeses demonstrated the presence of more than one amine. Notably, in 40% of these cheeses, the total concentration of BAs did not exceed 20 mg/kg, indicating relatively low levels of these substances. In 20% of the samples, total BA concentrations ranged from 27.78 to 44.05 mg/kg, while another 20% exhibited concentrations between 56.33 and 78.12 mg/kg. The remaining 20% of cheeses had significantly higher concentrations, ranging from 104.98 to 820.11 mg/kg. The highest total concentration of BAs, measured at 820.11 mg/kg, was found in Feta cheese (sample M).

The detection of BAs in the analyzed cheeses highlights the complex interplay between microbial populations and the potential for amine accumulation. In particular, the high TMCs and LAB counts in Feta cheeses suggest that some of the associated microorganisms may produce decarboxylases and thus significantly influence BA production. LAB are known to exhibit decarboxylase activity, which can lead to the formation of BAs such as TYR and PUT, especially in environments where enterococci are also present [44].

While the detection of BAs in cheese is often discussed in relation to microbial populations, it is equally important to consider the impact of hygienic and technological factors on their accumulation. Factors such as the heat treatment of milk, which can inactivate certain bacteria responsible for biogenic amine production, and sanitation practices during cheese production play a crucial role in controlling BA levels [21]. Additionally, parameters like pH, salt concentration, and ripening time significantly influence the enzymatic activity that leads to the formation of BAs [49]. By incorporating these perspectives, we can achieve a more comprehensive understanding of the factors contributing to BA accumulation in cheese.

In cheese production, elevated levels of TYR are commonly observed due to the metabolic activities of fermentative bacteria, particularly Lactobacillus and Enterococcus species. These bacteria facilitate the decarboxylation of the amino acid tyrosine, leading to the accumulation of TYR, a prevalent BA in various cheese types. The term “tyros”, derived from Greek, translates to cheese, underscoring the historical association between this BA and cheese fermentation processes [2,50]. Understanding the role of these bacteria in TYR production is essential, as the excessive consumption of TYR can pose health risks, particularly for individuals taking monoamine oxidase inhibitors (MAOIs) [51].

SPE was the second most frequently detected BA in white brine cheeses, highlighting its significant presence alongside TYR. This finding is particularly interesting given that SPE is less commonly reported in cheese compared to SPD. LAB, particularly those from the genera Lactobacillus and Enterococcus, are well-documented contributors to the synthesis of polyamines through the decarboxylation of precursor amino acids such as arginine and ornithine. These amino acids are converted to PUT, which subsequently serves as a precursor for SPE synthesis [52]. Once PUT is formed, it can be further modified through the action of spermidine synthase, which catalyzes the transfer of an aminopropyl group from decarboxylated S-adenosylmethionine (SAM) to PUT, resulting in the production of SPD. Subsequently, SPD can be converted to SPE through the addition of another aminopropyl group, again facilitated by spermidine synthase [53]. Additionally, the unique conditions of cheese production, including the specific microbial communities and the ripening environment, can influence SPE accumulation in the final product [2]. It is also important to note that the presence of SPE may not solely result from microbial activity; it can also originate from the milk used in cheesemaking, as polyamines can transfer from milk to cheese during fermentation [1]. SPE levels detected in this study underscore the complex interplay between microbial metabolism and the inherent properties of the milk, which together contribute to the BA profile of white brine cheeses.

The presence of enterococci, which can further contribute to the decarboxylation of amino acids, may exacerbate the accumulation of BA, particularly in cheeses with high microbial loads [20]. Notably, species such as *Enterococcus faecalis* and *Enterococcus faecium* have been identified as significant contributors to BA formation due to their decarboxylase activity [46]. Therefore, understanding the dynamics of these microbial populations is crucial for managing BA levels and ensuring the safety and quality of white brined cheeses.

### 3.3. Microorganisms Isolated from White Brined Cheeses with Biogenic Amine Production Potential

Decarboxylase activity was identified in 94 microorganisms isolated from white brined cheeses, as summarized in Table 2. The intensity of BA production under in vitro conditions varied based on the genera and species of the microorganisms and the set of isolates obtained from each cheese. None of the isolates demonstrated the capability to produce PHE or SPD. Approximately 57% of the isolates exhibited low levels of HIS production, ranging from 0.1 to 6.2 mg/L; however, *Lactiplantibacillus plantarum* (isolate M1) stood out with a HIS concentration of 30.3 mg/L in the growth medium. TRY was produced by about 56% of the isolates, although the concentrations remained relatively low, ranging from 1.3 to 14.1 mg/L.

Putrescine was detected in 71% of the tested microorganisms, with the majority producing between 0.5 and 90.4 mg/L. Elevated levels of PUT were observed in approximately 9% of these isolates, with concentrations ranging from 113.4 to 1282.1 mg/L. Significant PUT producers were identified as *Enterococcus durans*, *Bacillus licheniformis*, *Serratia marcescens*, *Klebsiella aerogenes*, and *Enterococcus faecium*. Furthermore, around 73% of isolates cultured in BHI broth enriched with BA precursors could produce cadaverine, albeit generally in low concentrations of 0.9–30.3 mg/L. Only one isolate, identified as *Acinetobacter lwoffii*, produced CAD above the threshold of 100 mg/L.

While spermine was the most frequently detected BA, tyramine and putrescine were the most abundant at elevated concentrations. SPE production capability was noted in 94% of isolates, while TYR production was observed in all isolates. *Enterococcus durans*, *Lacticaseibacillus casei*, *Levilactobacillus brevis*, and *Lactiplantibacillus plantarum* were identified as the most significant TYR producers, with concentrations in the culture medium ranging from 113.4 to 395.1 mg/L. Notably, no spermine-producing isolate exceeded 100 mg/L, with the highest SPE concentration recorded at 70.4 mg/L for *Enterococcus faecium* (isolate M7).

The capacity to produce BA at concentrations exceeding 200 mg/L was detected in fewer than 1% of the isolates. The microorganisms responsible for this high BA production were identified as *Levilactobacillus brevis*, *Enterococcus durans*, *Enterococcus faecium*, *Klebsiella aerogenes*, *Serratia marcescens*, and *Lacticaseibacillus casei*. All of these isolates were derived from various counter-sold Feta cheese samples.

The detection of BA at concentrations exceeding 200 mg/L in specific isolates from white brined cheeses raises significant concerns regarding food safety and quality. TYR and PUT were the most abundant BAs at these elevated levels, rather than HIS, which is often associated with adverse health effects. Additionally, other BAs, including PUT and CAD, can enhance the effects of HIS by competing for the same metabolic pathways, specifically those involving diamine oxidase (DAO) and monoamine oxidase (MAO) at the intestinal level [52,54]. The presence of microorganisms capable of producing high concentrations of TYR and PUT, such as *Levilactobacillus brevis* and *Enterococcus durans*, suggests that specific strains may thrive in the cheese environment, potentially leading to increased amine accumulation during storage and ripening [1,48]. Therefore, monitoring and controlling the populations of these biogenic amine-producing microorganisms is crucial to mitigate the risks associated with their consumption and to ensure the safety of white brined cheeses [2,46]. The determination of toxic levels of total BAs in foods is difficult due to various factors acting on their specification. Common levels of BAs in foodstuffs and beverages (levels < 100 mg/kg) do not represent a serious risk for healthy consumers because they are metabolized by detoxication enzymes in human intestines. This limit was exceeded for four cheeses. It is therefore recommended to monitor and control BA-producing microorganisms. These recommendations include strategies such as using non-biogenic amine-producing starter cultures, improving hygiene practices, and adjusting ripening conditions to keep the resulting biogenic amine content of the cheese as low as possible [17].

## 4. Conclusions

In this study, a comprehensive analysis of biogenic amines in white brined cheeses was conducted, highlighting the microbial diversity and potential implications for food safety and quality. The presence of BAs and polyamines was confirmed in 20 samples, with tyramine and spermine identified as the most abundant compounds. Notably, in three Feta samples, TYR concentrations exceeded 100 mg/kg, raising concerns regarding the health risks associated with high levels of this BA.

The analysis revealed that 94 microorganisms isolated from the cheeses exhibited decarboxylase activity, with significant producers identified, including *Levilactobacillus brevis* and *Enterococcus durans*. The correlation between high TMCs and LAB counts in some samples suggests that these microorganisms play a key role in BA production, particularly in association with specific strains of enterococci with decarboxylase activity rather than with total LAB. These findings underscore the importance of monitoring specific microbial populations in cheese production to mitigate the risks associated with biogenic amine accumulation.

This research emphasizes the need for the ongoing surveillance of BAs in white brined cheeses to ensure consumer safety and maintain product quality. Future studies should focus on identifying specific strains responsible for high BA production and developing effective strategies to control their levels during cheese production and storage. To mitigate the risks associated with biogenic amines, it is important to follow hygienic practices and production technologies along with microbial monitoring, as well as EFSA recommendations.

Due to adverse effects on health, BA accumulation in foods should be prevented [16], and it is important to develop new and effective strategies to eliminate these substances. Several approaches have been suggested, such as inhibiting bacteria that produce BAs, reducing the number of BA producers using the heat treatment of raw milk for cheese production, and controlling food microbiota applied. Applying starter cultures with amine oxidase activity is important, as these inhibit the formation of BAs.

## Figures and Tables

**Table 1:** Biogenic amines (BAs) and their concentrations in white brined cheeses (mg·kg^−1^); “ND” not detected. A, C, E, J, K—Balkan cheeses in consumer packaging; B, D, F, L—Balkan cheeses, counter-sold; M, N, O—Feta, counter-sold; P, Q, R—Jadel in consumer packaging; T—Jadel, counter-sold; V, EE—Akawi, gyros flavor; W—Akawi, garlic flavor; U—Akawi, tzatziki flavor. Limit of detection (LOQ): 0.07–0.10 mg·kg^−1^.

Sample ID	HIS	TYR	PUT	CAD	SPE	Total BAs
A	ND	21.08 ± 0.87	ND	ND	40.39 ± 2.63	61.47 ± 2.77
C	ND	20.54 ± 1.76	ND	ND	57.58 ± 2.50	78.12 ± 3.06
E	ND	15.70 ± 0.89	ND	ND	44.24 ± 2.11	59.94 ± 2.29
J	ND	3.99 ± 0.24	ND	ND	12.72 ± 0.83	16.71 ± 0.86
K	ND	3.84 ± 0.33	ND	ND	12.86 ± 0.73	16.70 ± 0.80
B	ND	7.39 ± 0.16	ND	ND	19.39 ± 1.91	26.78 ± 1.92
D	ND	2.38 ± 0.25	ND	ND	13.86 ± 0.87	16.24 ± 0.91
F	ND	3.23 ± 0.23	ND	ND	16.63 ± 0.96	19.86 ± 0.99
L	ND	ND	ND	ND	11.26 ± 0.56	11.26 ± 0.56
M	26.76 ± 2.11	380.43 ± 21.90	308.50 ± 19.29	31.01 ± 1.66	73.41 ± 1.77	820.11 ± 29.36
N	ND	122.71 ± 9.57	349.99 ± 0.91	ND	12.96 ± 1.84	485.66 ± 9.79
O	ND	133.25 ± 10.99	12.27 ± 0.67	ND	42.33 ± 2.05	187.85 ± 11.20
P	ND	7.71 ± 0.28	ND	ND	11.90 ± 0.67	19.61 ± 0.73
Q	ND	9.09 ± 0.60	ND	ND	18.71 ± 0.85	27.80 ± 1.04
R	ND	11.43 ± 0.86	ND	11.65 ± 0.65	20.97 ± 1.13	44.05 ± 1.56
T	ND	27.57 ± 1.88	ND	3.22 ± 0.09	5.04 ± 0.47	33.83 ± 1.94
V	ND	3.84 ± 0.33	ND	ND	12.86 ± 0.73	16.70 ± 0.80
EE	ND	41.98 ± 2.79	ND	3.45 ± 0.17	10.90 ± 0.65	56.33 ± 2.87
W	ND	ND	ND	ND	11.26 ± 0.56	11.26 ± 0.56
U	ND	ND	ND	ND	58.32 ± 3.40	104.98 ± 3.40

**Table 2:** Biogenic amine production by isolates from white brined cheeses (mg·L^−1^); “ND” not detected. Limit of detection (LOQ): 0.07–0.10 mg·L^−1^.

Cheese Sample	Isolate ID	Identified Microorganism	HIS	TYR	TRY	PUT	CAD	SPE	Total BAs
Balkan cheese—consumer packaging	A1	*Lactococcus lactis*	0.60 ± 0.04	2.80 ± 0.17	3.70 ± 0.19	0.80 ± 0.06	2.30 ± 0.12	2.50 ± 0.15	12.70 ± 0.32
	A2	*Lacticaseibacillus casei*	2.70 ± 0.16	81.70 ± 3.27	4.60 ± 0.32	10.70 ± 0.75	4.90 ± 0.25	32.70 ± 0.98	137.30 ± 3.52
	A5	*Staphylococcus equorum*	3.90 ± 0.16	47.53 ± 1.43	ND	5.60 ± 0.34	29.10 ± 0.87	43.57 ± 3.05	129.70 ± 3.50
	C1	*Lacticaseibacillus casei*	0.30 ± 0.01	41.20 ± 2.47	3.40 ± 0.20	1.40 ± 0.04	1.40 ± 0.04	0.10 ± 0.01	47.80 ± 2.48
	C3	*Acinetobacter lwoffii*	ND	38.82 ± 1.16	ND	2.70 ± 0.19	124.93 ± 8.75	37.55 ± 3.00	204.00 ± 9.32
	E1	*Lacticaseibacillus casei*	0.10 ± 0.01	21.40 ± 1.93	2.10 ± 0.13	1.60 ± 0.11	2.00 ± 0.16	30.50 ± 2.44	57.70 ± 3.12
	E2	*Serratia marcescens*	0.60 ± 0.05	13.50 ± 0.95	4.90 ± 0.34	1.30 ± 0.07	2.00 ± 0.08	33.10 ± 2.32	55.40 ± 2.53
	E3	*Lacticaseibacillus casei*	3.00 ± 0.18	1.80 ± 0.09	5.10 ± 0.26	35.50 ± 2.49	10.80 ± 0.32	32.00 ± 1.28	88.20 ± 2.83
	E5	*Staphylococcus warneri*	2.00 ± 0.08	1.40 ± 0.13	11.20 ± 0.45	4.30 ± 0.39	2.30 ± 0.09	0.40 ± 0.01	21.60 ± 0.62
	E6	*Staphylococcus equorum*	1.70 ± 0.15	2.10 ± 0.06	10.30 ± 0.72	4.50 ± 0.41	2.50 ± 0.23	ND	21.10 ± 0.87
	E8	*Kocuria varians*	ND	18.82 ± 0.94	ND	ND	ND	25.10 ± 2.01	43.92 ± 2.22
	G2 *	*Kocuria* sp.	0.40 ± 0.03	0.90 ± 0.07	2.90 ± 0.26	0.80 ± 0.05	0.90 ± 0.05	0.10 ± 0.01	6.00 ± 0.28
	H7	*Serratia marcescens*	ND	5.50 ± 0.50	ND	ND	ND	20.44 ± 1.02	25.94 ± 1.14
	J3	*Kocuria* sp.	0.40 ± 0.03	4.70 ± 0.19	2.90 ± 0.26	3.80 ± 0.11	1.50 ± 0.05	0.30 ± 0.02	13.60 ± 0.35
	J4	*Bacillus cereus*	ND	4.30 ± 0.13	ND	ND	ND	18.03 ± 0.90	22.33 ± 0.91
	J5	*Enterococcus durans*	ND	4.43 ± 0.35	ND	ND	8.99 ± 0.72	24.43 ± 1.71	37.85 ± 1.89
	J8	*Enterococcus faecium*	ND	8.06 ± 0.40	ND	ND	ND	23.23 ± 0.70	31.29 ± 0.81
	J9	*Enterococcus durans*	ND	2.33 ± 0.19	ND	ND	6.47 ± 0.19	16.53 ± 1.16	25.33 ± 1.19
	K1	*Lactococcus lactis*	ND	5.24 ± 0.26	ND	ND	ND	22.21 ± 1.78	27.45 ± 1.80
	K2	*Lacticaseibacillus paracasei*	ND	1.48 ± 0.07	ND	ND	ND	20.22 ± 1.82	21.70 ± 1.82
Balkan cheese—counter-sold	B3	*Lactiplantibacillus plantarum*	0.30 ± 0.03	1.50 ± 0.12	3.50 ± 0.28	0.60 ± 0.02	1.50 ± 0.14	20.80 ± 1.66	28.20 ± 1.70
	B4	*Lactococcus cremoris*	0.20 ± 0.01	5.10 ± 0.46	1.30 ± 0.09	1.50 ± 0.14	1.30 ± 0.04	30.20 ± 2.11	39.60 ± 2.17
	D4	*Acinetobacter calcoaceticus*	1.60 ± 0.11	1.80 ± 0.13	14.10 ± 0.42	6.80 ± 0.54	2.50 ± 0.20	10.40 ± 0.73	37.20 ± 1.04
	D5	*Lactococcus lactis*	ND	20.14 ± 1.81	ND	ND	ND	17.35 ± 1.39	37.49 ± 2.28
	F1	*Enterococcus faecium*	0.30 ± 0.02	1.40 ± 0.10	2.30 ± 0.14	1.20 ± 0.08	1.00 ± 0.03	5.10 ± 0.26	11.30 ± 0.32
	F3	*Bacillus amyloliquefacinns*	0.50 ± 0.04	1.60 ± 0.05	1.80 ± 0.14	0.80 ± 0.07	1.40 ± 0.10	10.50 ± 0.32	16.60 ± 0.37
	F4	*Staphylococcus hominis*	0.40 ± 0.02	5.20 ± 0.16	2.60 ± 0.18	0.80 ± 0.07	1.80 ± 0.05	8.40 ± 0.59	19.20 ± 0.64
	F5	*Bacillus licheniformis*	0.60 ± 0.03	2.90 ± 0.20	4.50 ± 0.27	2.00 ± 0.16	1.60 ± 0.13	10.30 ± 0.82	21.90 ± 0.91
	I1	*Lysinibacillus fusiformis*	0.70 ± 0.06	2.10 ± 0.15	3.70 ± 0.26	1.00 ± 0.05	2.40 ± 0.17	ND	9.90 ± 0.35
	I2	*Bacillus horneckiae*	0.60 ± 0.04	1.80 ± 0.09	4.40 ± 0.35	1.10 ± 0.04	2.40 ± 0.19	ND	10.30 ± 0.41
	L1	*Lactiplantibacillus plantarum*	ND	4.30 ± 0.39	ND	ND	ND	15.95 ± 1.12	20.25 ± 1.18
	L5	*Lacticaseibacillus casei*	ND	1.30 ± 0.12	ND	ND	ND	19.30 ± 1.54	20.60 ± 1.55
Feta—counter-sold	M1	*Lactiplantibacillus plantarum*	30.30 ± 1.21	152.30 ± 13.71	3.40 ± 0.10	0.90 ± 0.08	1.70 ± 0.14	2.40 ± 0.22	191.00 ± 13.76
	M2	*Levilactobacillus brevis*	0.30 ± 0.02	201.90 ± 12.11	4.00 ± 0.32	0.70 ± 0.04	2.20 ± 0.15	62.50 ± 2.50	271.60 ± 12.37
	M4	*Enterococcus durans*	6.20 ± 0.50	10.50 ± 0.53	8.10 ± 0.49	1282.10 ± 89.75	30.30 ± 0.91	2.90 ± 0.17	1340.10 ± 89.76
	M7	*Enterococcus faecium*	6.00 ± 0.18	2.00 ± 0.14	12.10 ± 0.48	395.80 ± 19.79	20.30 ± 1.83	70.40 ± 2.11	506.60 ± 19.99
	N1	*Klebsiella aerogenes*	0.30 ± 0.01	1.70 ± 0.07	2.40 ± 0.10	199.80 ± 15.98	1.60 ± 0.10	0.40 ± 0.02	206.20 ± 15.98
	N2	*Staphylococcus warneri*	0.40 ± 0.02	2.10 ± 0.17	2.60 ± 0.08	1.90 ± 0.13	1.20 ± 0.07	1.00 ± 0.09	9.20 ± 0.26
	N4	*Serratia marcescens*	ND	37.25 ± 1.12	ND	201.60 ± 6.05	9.80 ± 0.88	34.43 ± 1.03	283.08 ± 6.30
	N6	*Lacticaseibacillus casei*	1.60 ± 0.05	395.10 ± 23.71	9.80 ± 0.49	6.40 ± 0.38	2.70 ± 0.19	2.70 ± 0.24	418.30 ± 23.72
	O1	*Staphylococcus warneri*	0.40 ± 0.03	4.70 ± 0.42	2.90 ± 0.26	1.90 ± 0.13	1.90 ± 0.17	2.40 ± 0.12	14.20 ± 0.56
	O3	*Kocuria* sp.	0.40 ± 0.01	1.20 ± 0.11	3.20 ± 0.16	1.70 ± 0.15	1.60 ± 0.14	30.20 ± 1.51	38.30 ± 1.54
	O4	*Psychrobacillus psychrodurans*	0.30 ± 0.01	81.20 ± 5.68	3.40 ± 0.24	1.40 ± 0.08	1.40 ± 0.04	8.10 ± 0.73	95.80 ± 5.74
	O5	*Lacticaseibacillus casei*	0.30 ± 0.02	2.00 ± 0.16	1.70 ± 0.07	1.70 ± 0.07	1.40 ± 0.11	7.80 ± 0.39	14.90 ± 0.45
	O7	*Enterococcus durans*	2.00 ± 0.16	113.40 ± 10.21	ND	113.40 ± 7.94	ND	40.70 ± 2.44	269.50 ± 13.16
	O8	*Lactococcus cremoris*	0.30 ± 0.02	2.30 ± 0.16	1.80 ± 0.09	1.50 ± 0.14	1.60 ± 0.06	0.60 ± 0.02	8.10 ± 0.24
	O9	*Enterococcus faecium*	1.60 ± 0.05	86.40 ± 3.46	ND	ND	ND	4.30 ± 0.34	92.30 ± 3.47
Jadel—consumer packaging	P3	*Lactococcus lactis*	0.40 ± 0.04	15.60 ± 0.47	2.60 ± 0.16	1.00 ± 0.04	1.50 ± 0.09	20.20 ± 0.81	41.30 ± 0.95
	Q2	*Klebsiella aerogenes*	0.30 ± 0.02	5.00 ± 0.20	2.80 ± 0.14	1.00 ± 0.09	1.90 ± 0.11	10.10 ± 0.30	21.10 ± 0.42
	Q3	*Staphylococcus equorum*	0.50 ± 0.04	6.30 ± 0.57	3.10 ± 0.22	3.70 ± 0.19	2.00 ± 0.06	8.10 ± 0.41	23.70 ± 0.76
	Q5	*Kocuria varians*	3.10 ± 0.12	0.20 ± 0.01	3.10 ± 0.22	1.70 ± 0.07	1.20 ± 0.08	4.20 ± 0.21	13.50 ± 0.34
	R4	*Bacillus licheniformis*	0.40 ± 0.02	1.70 ± 0.10	3.00 ± 0.27	1.30 ± 0.10	1.80 ± 0.09	20.80 ± 0.62	29.00 ± 0.70
	R6	*Lactiplantibacillus plantarum*	ND	23.61 ± 1.18	ND	ND	ND	18.90 ± 1.51	42.51 ± 1.92
	T1 **	*Lactococcus lactis*	ND	17.98 ± 0.72	ND	ND	8.70 ± 0.78	23.38 ± 1.17	50.06 ± 1.58
Akawi—unflavored, stored for 1 year at 20–25 °C	Z1	*Bacillus licheniformis*	ND	15.51 ± 1.40	ND	8.51 ± 0.60	1.76 ± 0.12	10.69 ± 0.32	36.47 ± 1.56
	Z3	*Lactococcus lactis*	ND	39.61 ± 1.19	ND	4.28 ± 0.30	1.92 ± 0.15	26.98 ± 1.62	72.79 ± 2.04
	AA4	*Enterococcus durans*	ND	9.07 ± 0.27	ND	13.07 ± 1.05	5.41 ± 0.27	14.58 ± 0.73	42.13 ± 1.33
	AA5	*Enterococcus faecalis*	ND	12.38 ± 0.87	ND	ND	ND	28.19 ± 1.13	40.57 ± 1.42
	BB1	*Lactiplantibacillus plantarum*	ND	16.38 ± 1.15	ND	ND	ND	44.37 ± 3.55	60.75 ± 3.73
	BB6	*Lacticaseibacillus racasei*	ND	21.51 ± 1.29	ND	ND	ND	39.36 ± 1.18	60.87 ± 1.75
	CC1	*Enterococcus durans*	ND	17.08 ± 1.54	ND	14.68 ± 0.73	7.16 ± 0.50	27.72 ± 2.22	66.64 ± 2.84
	CC3	*Enterococcus faecium*	ND	15.00 ± 0.60	ND	11.07 ± 0.33	5.41 ± 0.32	22.26 ± 2.00	53.74 ± 2.14
	CC6	*Enterococcus durans*	ND	18.53 ± 1.11	ND	12.10 ± 1.09	5.77 ± 0.23	28.93 ± 1.45	65.33 ± 2.14
	CC7	*Lacticaseibacillus casei*	ND	15.50 ± 0.93	ND	11.80 ± 0.59	6.06 ± 0.24	25.77 ± 1.55	59.13 ± 1.91
	DD2	*Lacticaseibacillus casei*	ND	13.20 ± 1.06	ND	14.62 ± 1.32	6.77 ± 0.54	22.56 ± 1.13	57.15 ± 2.10
	DD4	*Lactiplantibacillus paraplantarum*	ND	18.41 ± 1.29	ND	ND	ND	34.14 ± 2.05	52.55 ± 2.42
Akawi—gyros flavor	V1	*Klebsiella oxytoca*	0.50 ± 0.04	12.40 ± 0.87	4.50 ± 0.32	2.30 ± 0.18	2.00 ± 0.18	2.40 ± 0.22	24.10 ± 0.98
	V3	*Bacillus licheniformis*	4.70 ± 0.42	2.10 ± 0.17	4.60 ± 0.41	3.50 ± 0.21	5.80 ± 0.23	3.20 ± 0.29	23.90 ± 0.75
	V4	*Staphylococcus hominis*	0.70 ± 0.06	11.10 ± 1.00	3.90 ± 0.31	2.90 ± 0.23	1.90 ± 0.15	2.80 ± 0.11	23.30 ± 1.09
	V5	*Kocuria varians*	0.50 ± 0.05	3.30 ± 0.23	4.10 ± 0.33	0.50 ± 0.03	1.80 ± 0.14	2.80 ± 0.14	13.00 ± 0.45
	EE1	*Lactiplantibacillus plantarum*	ND	21.14 ± 0.63	10.90 ± 0.33	6.30 ± 0.19	2.90 ± 0.09	37.26 ± 1.49	78.50 ± 1.67
	EE2	*Lentilactobacillus hilgardii*	ND	19.61 ± 0.59	ND	ND	ND	43.96 ± 3.96	63.57 ± 4.00
	EE5	*Enterococcus durans*	ND	16.31 ± 1.14	ND	90.41 ± 8.14	5.44 ± 0.49	26.54 ± 1.33	138.70 ± 8.34
	EE6	*Bacillus cereus*	ND	18.59 ± 0.56	ND	ND	ND	39.74 ± 3.18	58.33 ± 3.23
	EE7	*Staphylococcus warneri*	ND	27.01 ± 1.89	ND	ND	ND	50.40 ± 1.51	77.41 ± 2.42
	EE8	*Enterococcus faecium*	ND	31.20 ± 1.56	ND	ND	ND	32.81 ± 2.62	64.01 ± 3.05
	EE9	*Enterococcus durans*	ND	24.77 ± 1.24	ND	ND	ND	41.34 ± 2.89	66.11 ± 3.15
Akawi—garlic flavor	W1	*Bacillus cereus*	0.30 ± 0.02	1.10 ± 0.07	2.80 ± 0.08	0.60 ± 0.04	1.10 ± 0.09	12.10 ± 0.73	18.00 ± 0.74
	W2	*Staphylococcus warneri*	0.30 ± 0.01	1.50 ± 0.06	3.10 ± 0.25	1.60 ± 0.14	1.20 ± 0.10	9.70 ± 0.87	17.40 ± 0.93
	W3	*Enterococcus faecium*	0.30 ± 0.01	1.10 ± 0.04	1.40 ± 0.08	1.70 ± 0.05	1.20 ± 0.06	12.70 ± 0.76	18.40 ± 0.77
	W4	*Enterococcus faecalis*	0.20 ± 0.01	2.00 ± 0.16	1.50 ± 0.05	4.20 ± 0.38	1.00 ± 0.06	4.30 ± 0.22	13.20 ± 0.47
Akawi—tzatziki flavor	U1	*Enterococcus durans*	ND	16.71 ± 1.50	ND	ND	ND	34.11 ± 1.36	50.82 ± 2.03
	U2	*Lacticaseibacillus paracasei*	ND	40.02 ± 3.20	ND	6.04 ± 0.18	5.64 ± 0.45	26.33 ± 1.05	78.03 ± 3.41
	U3	*Lactococcus lactis*	ND	17.25 ± 1.38	ND	ND	ND	34.47 ± 2.07	51.72 ± 2.49
	U4	*Bacillus safensis*	ND	43.94 ± 1.76	7.02 ± 0.42	4.51 ± 0.32	9.57 ± 0.38	36.77 ± 1.84	101.81 ± 2.63
	U5	*Enterococcus durans*	ND	16.83 ± 1.35	ND	ND	ND	42.18 ± 2.53	59.01 ± 2.87
	U8	*Enterococcus faecium*	ND	14.05 ± 0.42	ND	ND	ND	29.99 ± 1.20	44.04 ± 1.27
	U9	*Bacillus licheniformis*	1.70 ± 0.14	1.80 ± 0.11	4.70 ± 0.42	490.80 ± 44.17	5.10 ± 0.36	3.60 ± 0.29	507.70 ± 44.18
	X1	*Lactococcus lactis*	0.60 ± 0.04	1.80 ± 0.14	4.40 ± 0.22	1.10 ± 0.03	2.20 ± 0.09	ND	10.10 ± 0.28
	X2	*Lactococcus cremoris*	0.70 ± 0.03	1.70 ± 0.10	4.00 ± 0.16	1.70 ± 0.12	1.90 ± 0.11	ND	10.00 ± 0.25
	X3	*Staphylococcus epidermidis*	0.70 ± 0.04	1.50 ± 0.06	3.30 ± 0.10	1.30 ± 0.12	1.60 ± 0.14	ND	8.40 ± 0.22
	X4	*Lacticaseibacillus paracasei*	0.70 ± 0.04	1.40 ± 0.11	3.70 ± 0.22	1.30 ± 0.12	1.80 ± 0.16	1.60 ± 0.14	10.50 ± 0.35
	X5	*Lactiplantibacillus paraplantarum*	0.80 ± 0.07	1.20 ± 0.11	4.40 ± 0.31	1.40 ± 0.11	2.10 ± 0.13	2.50 ± 0.23	12.40 ± 0.44

* Balkan cheese in customer packaging with spicy flavoring. ** Jadel—counter-sold.

## Data Availability

The original contributions presented in this study are included in the article. Further inquiries can be directed to the corresponding author.
